# Chronic Viral Infections and Al Amyloidosis: An Uncommon Association

**DOI:** 10.1002/ccr3.9616

**Published:** 2025-04-02

**Authors:** Alberto Palladini, Gabriele Cusumano, Ottavio Martellucci, Antonietta Gigante, Gabriella D'Ettorre, Maria Teresa Petrucci, Maurizio Muscaritoli, Mariangela Palladino

**Affiliations:** ^1^ Department of Translational and Precision Medicine Sapienza University of Rome Rome Italy; ^2^ Department of Public Health and Infectious Disease Sapienza University of Rome Rome Italy; ^3^ Department of Cellular Biotechnology and Hematology Sapienza University of Rome Rome Italy

**Keywords:** amyloidosis, immune system, inflammation, viral infections

## Abstract

AL‐amyloidosis is caused by deposition of pathological insoluble proteins in organs and tissues. HIV infection is a multi‐system disorder causing chronic inflammation. We report a case of AL‐amyloidosis associated with HIV suggesting that HIV‐associated immune dysregulation might be critical for pathogenesis of plasma cells dyscrasias and then AL amyloidosis.

**Summary**

- AL amyloidosis is caused by deposition of pathological insoluble proteins in organs and tissues.
- HIV infection is a multi‐system disorder causing chronic inflammation.
- We report a case of AL amyloidosis associated with HIV suggesting that HIV‐associated immune dysregulation might be critical for pathogenesis of plasma cells dyscrasias and then AL amyloidosis.

## 1 Introduction

Systemic amyloid A (AA) amyloidosis is usually secondary to various chronic infectious, while amyloid light‐chain (AL) amyloidosis is associated with plasma cell dyscrasia. In the literature, only few studies report correlations between infections and AL amyloidosis. We report an unusual case of AL amyloidosis developing in the setting of chronic hepatitis B and HIV infections.

Our clinical case suggests a link between chronic infections and eventual onset of AL amyloidosis. There is suspicion that AL amyloidosis is linked to systemic inflammation of the body, but more research is needed to confirm this. Furthermore, the case was a great therapeutic challenge, based on the balance between antiviral and immunosuppressive therapy.

## 2 Case History

We present a case of a caucasian‐57‐year‐old man recently presenting to the emergency department (ED) sent by his family doctor referring fever with night sweats, asthenia for mild activities (New York Heart Association—NYHA III) and itching from 1 month. Autonomously, the patient performed blood tests that revealed proteinuria (1 g/L) and monoclonal spike in β1 zone on serum electrophoresis. In the clinical history, the patient reported: tooth extractions 1 month before, hepatitis B, cholecystectomy for gallstones, discectomy for a herniated disc, gastroesophageal reflux, and rib fractures due to childhood trauma. He referred to smoking and alcohol use for 20 years. In the ED patient's arterial blood pressure (ABP) was 100/70 mmHg and heart rate was 97 bpm. The remaining vital signs were normal. The physical examination showed excoriation and scratch injuries of the skin in the arms and legs and pitting edema and hyperemia in the legs.

## 3 Methods

**FIGURE 1:**
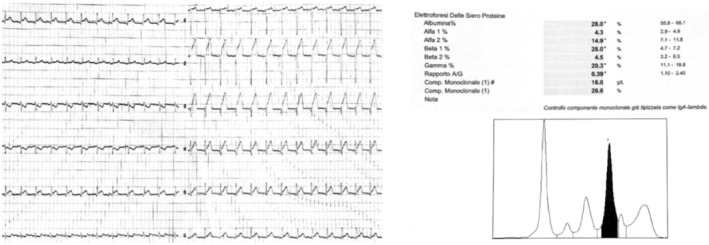
(left) Tipical low QRS voltages are common in cardiac involvement. (right) Serum electrophoresis showing β1 peak.

**FIGURE 2:**
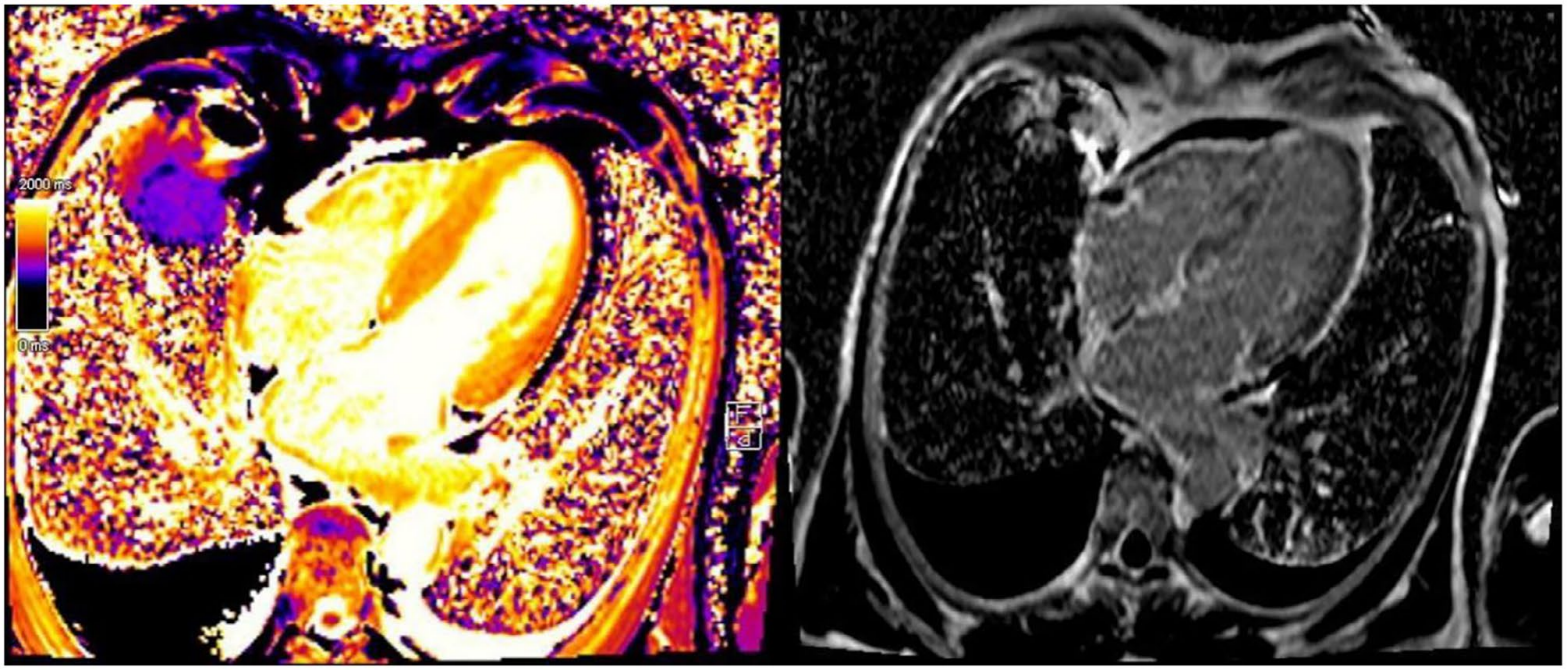
(left) Cardiac MRI showing hypertrophic cardiomyopathy and thickened interventricular septum. (right) T1 relaxation time maps. It can be seen: Diffuse fibrotic tissue, pleural and pericardial effusion.

**FIGURE 3:**
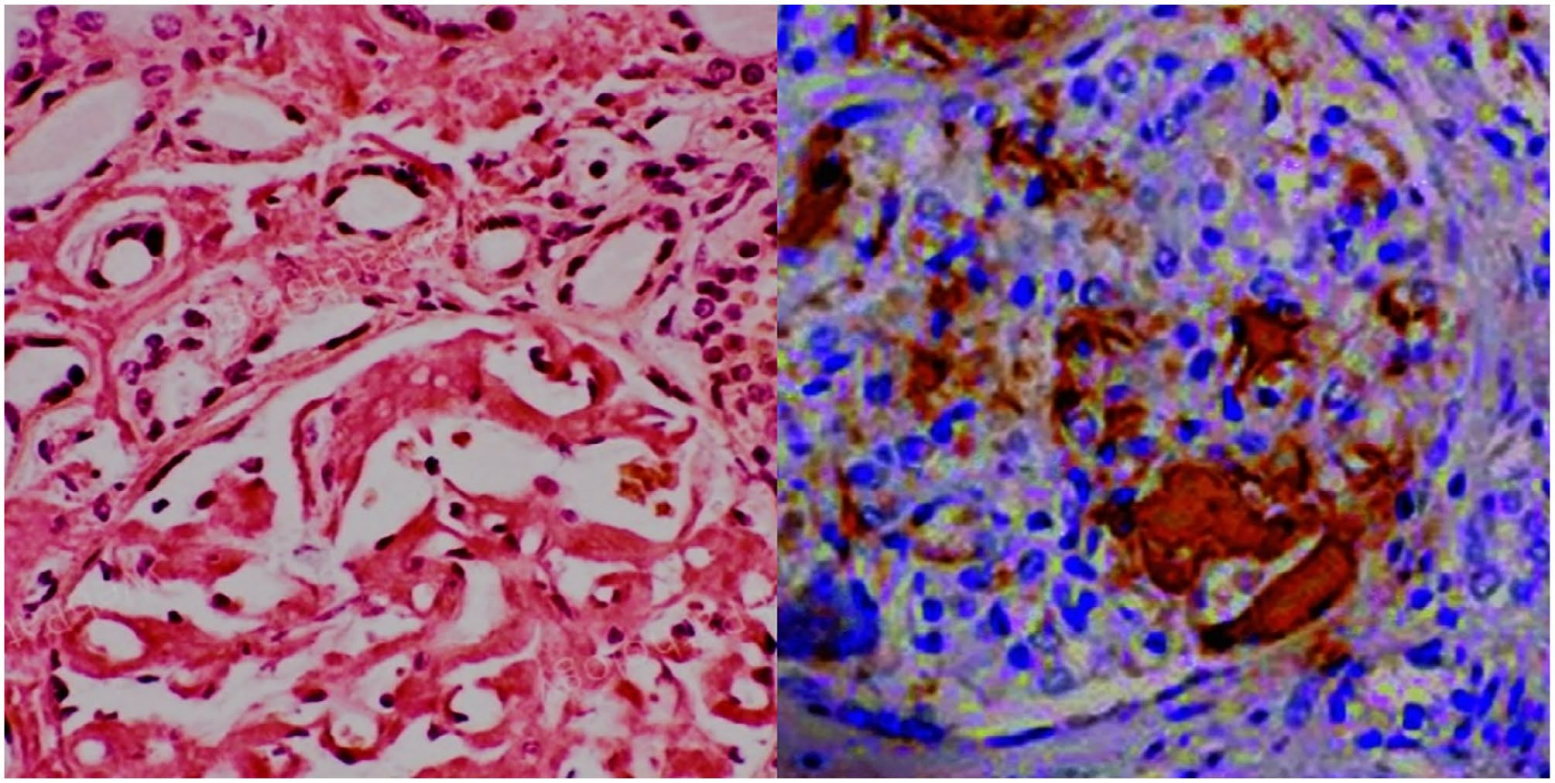
(left) Positive Congo red stain in renal biopsy. (right) Immunohistochemical stain showing positive staining with antibody against lambda light chains.

Blood exams revealed increased levels of serum aspartate aminotransferase (AST), alanine aminotransferase (ALT), gamma‐glutamyl transferase (GGT), lactate dehydrogenase (LDH), D‐Dimer, high‐sensitivity troponin (stable after 3 h). Arterial blood gas analysis was normal and Severe Acute Respiratory Syndrome COronaVirus 2 (SARS Cov‐2 RNA) testing of the swab was negative. Chest X‐Ray documented thickened smoothly interlobular septae in the right parahilar, subtle parenchymal hypodiaphania on the left basal area and blunting of the costophrenic angles. After blood cultures and swabs collection for *St. Aureus Methicillin‐Resistant*, empiric antibiotic therapy with piperacillin/tazobactam and daptomycin was then started. Compression ultrasonography of lower extremity outlined partial and segmental incompressibility of the right popliteal vein compatible with subacute deep vein thrombosis (DVT) with partial recanalization. DVT treatment with low‐molecular‐weight heparin was immediately started. Upon admission to our Unit the patient was in fair clinical condition. His heart rate was still 95 bpm and ABP was 110/60 mmHg. On physical examination, grade 2/6 systolic murmur localized to the area of the left sternal border and bilateral basal crackles were documented; vesicular breath sounds were diminished. Further blood exams showed hypoalbuminemia (15 g/L), hypoproteinemia (50 g/L) and decreased levels of antithrombin III (< 60%). Electrocardiography showed low QRS voltage in precordial leads while echocardiography documented hypertrophic cardiomyopathy and thickened interventricular septum. Serum protein electrophoresis showed a band in β1 globulin region; on immunofixation electrophoresis, serum revealed IgA lambda monoclonal component (16 g/L) **(**Figure 1 **)**. The 24‐h proteinuria was 6.4 g and Bence‐Jones protein was detected in the urine. Serum IgA was higher than normal range (12,75 g/L). Total body computed tomography (CT) scan showed bilateral pleural effusion and small pericardial effusion (6 mm). In the suspicion of amyloidosis, despite the negativity of the abdominal fat pad fine‐needle aspiration biopsy, myocardial scintigraphy and magnetic resonance imaging (MRI) were then performed. Scintigraphy (and genetic testing) was negative for the transthyretin amyloidosis (ATTR) subtype. Cardiac MRI revealed asymmetric, non‐obstructive and hypertrophic cardiomyopathy, thickened interventricular septum (20 mm), and circumferential late gadolinium enhancement in the sub‐mesocardial area, indicating amyloid accumulation (Figure 2). Finally, kidney biopsy showed Congo red positivity on light microscopy; while immunohistochemistry for anti‐lambda antibodies was positive, confirming light‐chain (AL) amyloidosis (Figure 3).

Based on the patient's anamnesis, reporting unprotected sex and recurring fever over the last few months, microbiological investigations were performed, revealing chronic hepatitis B infection (serum HBV DNA level of > 95 million copies/ml), Epstein Barr virus (EBV) DNA infection and HIV infection (serum HIV RNA level of > 2 million copies/ml). Lymphocyte subpopulation analysis showed lower CD4+ (208/μL), and CD4/CD8 ratio of 0.10. Quantiferon‐TB Gold was negative.

## 4 Follow‐Up

Bictegravir/emtricitabine/tenofovir alafenamide treatment was immediately started. Dermatologist consultation for evaluation of desquamative‐erythematous and itchy skin lesions, diagnosed as mild to moderate psoriasis vulgaris, and recommended topical therapy with calcipotriol and betamethasone. The patient was then discharged in mild clinical condition with outpatient cardiological and internal medicine follow‐up within 10 days after discharge. Cardiac therapy at discharge was based on furosemide 50 mg and bisoprolol 2.5 mg daily. Moreover, a bone marrow biopsy was scheduled. At the follow‐up visit, patient lower limbs edema had worsened and high‐grade proteinuria was stable. Spironolactone 50 mg/day and losartan 12.5 mg/day were then prescribed. Due to persistent mild hypotension (95/60 mmHg), losartan and spironolactone were soon suspended, furosemide dosage was increased up to 150 mg/day and dapaglifozin 10 mg was then started with subsequent significant decrease in lower limbs edema. One month after discharge, doppler ultrasound of lower limbs showed resolution of DVT. Bone marrow biopsy revealed 30% CD138+, CD20−, CD56±, D1 cyclin‐ monoclonal plasma cells with no sign of amyloid infiltration expressing lambda light chain. According to International Myeloma Working Group (IMWG) updated criteria for the diagnosis of multiple myeloma, diagnosis of plasma cell dyscrasias were excluded [1]. After obtaining viral load suppression, the patient is going to start bortezomib‐based induction therapy plus daratumumab.

## 5 Discussion

Amyloidoses are a typology of rare diseases characterized by the accumulation of protein fibrillary aggregates in heart, kidney, gastrointestinal tract, peripheral and autonomic nerves, skin, joints and blood vessels of all tissues [2]. The proteins typically are antiparallel β‐sheets, which show X‐Ray diffraction, orange‐red appearance under light microscopy and apple‐green birefringence under polarized light [3, 4]. Primary or light chain (AL) amyloidosis is the most frequent type of systemic amyloidosis. AL is caused by abnormal proliferation of resident bone marrow monoclonal plasma cells producing unstable light chains. Lambda subtype accounts for 75% of all cases. Those amyloidogenic free light chains can then accumulate in tissues [5] evolving in progressive organ dysfunction. Typically, plasma cells are less than 10% at the bone marrow biopsy [6]. Symptoms depend on the organ involvement, ranging from heart failure with preserved ejection fraction, nephrotic syndrome, organomegaly (mainly tongue and salivary glands) to peripheral neuropathies and unspecific symptoms such as fatigue, asthenia and body weight loss. The most common cause of the death in patients with amyloidosis is heart failure [7]. Clinical suspicion of AL amyloidosis will require biopsy of the involved organs, preferably the fat pad or, more rarely, kidney, salivary gland, heart, and liver [8]. Currently, there are no clear guidelines on systemic AL amyloidosis treatment. However, since both AL amyloidosis and multiple myeloma are monoclonal plasma cell dyscrasias, AL amyloidosis treatment strategies and medications are derived from the anti‐plasma cell therapy used for multiple myeloma [9, 10]. In our case report, the patient presented non‐specific symptoms and signs (hypotension, severe asthenia, limitation in ordinary activity due to fatigue and dyspnea, itching, recurrent low‐grade fever, DVT). Laboratory tests showed nephrotic‐range proteinuria and monoclonal IgA lambda. The first clinical suspicion of infiltrative disease arose from the low QRS voltage in V1‐6 and limb leads. Amyloidosis electrocardiogram often shows QRS voltage less than 0.5 mm in the peripheral leads and less than 1 mm in precordial leads; the Sokolow/Lyon index which represents the sum of the S wave in V1 and R wave in V5 and V6 (SV1 + RV5/6) is usually < 1.5 mV and is typically found in 30%–70% of patients with AL amyloidosis [11]. Echocardiography usually documents hypertrophic cardiomyopathy, with no prior history of hypertension [12]. Cardiac MRI was suggestive of amyloid accumulation (Figure 2). Myocardial scintigraphy and genetic exams were even performed to rule out transthyretin (ATTR) amyloidosis. Despite cardiac MRI sensitivity and specificity is 85%–90% [12], the gold standard exam to diagnose amyloidosis is histological evidence of amyloid deposition in the tissues [13]. In our case, given the negativity of fat pad aspiration (58% sensitivity and 100% specificity in AL amyloidosis), cardiac involvement and high clinical suspicion, renal biopsy was mandatory to confirm diagnosis. HBV and HIV are typically associated with amyloid A (AA) amyloidosis [14]. Only few studies describe development of AL amyloidosis after HBV and HIV infection and there is no unique consensus about the etiopathogenesis [15]. The main hypotheses are that HBV is responsible for chronic immunological stimulation, while HIV antigens act like superantigens and stimulate B cell proliferation determining immunoglobulins production; on the other hand, HIV infection causes CD4+ T cell depletion, which induces immunodeficiency [16]. Immunoglobulins can misfold and accumulate as amyloid fibrils and lead to amyloidosis. Moreover, some studies correlate monoclonal proliferation of plasma cells and HIV [16, 17]. In particular, a higher prevalence rate of monoclonal gammopathy of undetermined significance (MGUS) has been found in HIV‐infected patients, ranging between 3% and 26% [18]. Furthermore, prevalence of MGUS is higher in newly diagnosed young (age onset between 18 and 40) HIV patients compared with the general population [19]. In these patients, MGUS usually recover after receiving antiviral therapy [20]. These data enforce the hypothesis that chronic antigenic stimulation leads to B cell hyperplasia and that B cell dysregulation in the setting of impaired T cell responses, could contribute to the clonal phenotype. AL Amyloidosis is usually secondary to plasma cell dyscrasias. At the best of our knowledge, there are neither previous case reports on systemic AL amyloidosis in HIV positive patients nor hypothesis about the underlying mechanisms that drive the onset of AL amyloidosis in HIV infection. The evolution of the HIV epidemic has induced new unsolved issues regarding HIV infection and its effects on the dysregulation of immune system. HIV chronically activates immune system generating also an early immunosenescence [21]. Many factors influence chronic immune activation. The increase in circulating activated lymphocytes with a high replication rate is followed by an increase in the profile of inflammatory cytokines Il‐1, IL‐6, TNF‐Alfa, and IFN –gamma [22]. Consequently, such abnormal cytokine production contributes to the impairment of cell‐mediated immunity and chronic immune activation.

## 6 Conclusion

Our clinical case represents a rare case of HIV and HBV‐associated AL amyloidosis with IgA lambda monoclonal component. Pathophysiological links between HIV, HBV, and AL amyloidosis need to be accurately studied. Our case report suggests that HIV‐associated immune dysregulation might be critical for pathogenesis of plasma cell dyscrasias and then AL amyloidosis. Thus, we hypothesize that early immune restoration in our patient is important to remodulate the activity of HIV‐induced immune system and, hopefully, to respond to anti‐plasma cell neoplasm therapy.

We believe that this unusual observation presented as clinical case might be important for infectious disease specialist, general practitioners, cardiologists, and nephrologists to rapidly recognize risk factors for AL amyloidosis and thus facilitate the diagnosis and to start specific treatment.

## Author Contributions

**Alberto Palladini:** conceptualization, data curation, formal analysis, investigation, methodology. **Gabriele Cusumano:** conceptualization, data curation, formal analysis, resources, writing – original draft. **Ottavio Martellucci:** conceptualization, data curation, formal analysis, investigation, methodology, writing – original draft. **Antonietta Gigante:** funding acquisition, supervision, validation. **Gabriella D'Ettorre:** methodology, visualization. **Maria Teresa Petrucci:** investigation, resources, validation. **Maurizio Muscaritoli:** funding acquisition. **Mariangela Palladino:** conceptualization, data curation, formal analysis, funding acquisition, investigation, methodology, project administration, resources, supervision, validation, visualization, writing – original draft.

## Ethics Statement

This article does not contain any studies with human participants or animals performed by any of the authors.

## Consent

Written informed consent has been taken from the patient for the use of his clinical data.

## Conflicts of Interest

The authors declare no conflicts of interest.

## Key Message

Chronic viral infections as potential risk factor for AL amyloidosis.

## Data Availability

The authors have nothing to report.
